# Association of DNA methylation-predicted GDF15 and frailty in older adults based on NHANES 1999–2002

**DOI:** 10.1097/MD.0000000000046816

**Published:** 2025-12-26

**Authors:** Meisheng Zou, Suhong Wu

**Affiliations:** a Department of Geriatrics, Zhongshan City People’s Hospital, Zhongshan City, China.

**Keywords:** DNA methylation, frailty, GDF15, NHANES database, older adults

## Abstract

GDF15 (growth differentiation factor 15) serves as a significant indicator across diverse physiological states and disease processes. Increased GDF15 concentrations are strongly implicated in heightened frailty risk. Nevertheless, the prognostic utility of DNA methylation (DNAm)-estimated GDF15 levels for frailty remains inadequately investigated. This research seeks to examine the association between DNAm-predicted GDF15 concentrations and frailty among older individuals residing in the United States. This investigation leveraged data from the 1999 to 2002 National Health and Nutrition Examination Survey, employing a cross-sectional design. This study included 1553 community-dwelling older adults. Weighted logistic regression was utilized, supplemented by sensitivity assessments, to evaluate the link between DNAm-predicted GDF15 concentrations and frailty in participants aged ≥60 years. The restricted cubic spline technique was applied to further characterize this association. Additionally, covariate-specific subgroup evaluations and interaction assessments were visualized using a forest plot. Elevated concentrations of GDF15 predicted by DNA methylation were statistically associated with greater frailty likelihood (odds ratio = 1.14, 95% confidence interval: 1.03–1.26). Individuals within the highest third tertile of DNAm-predicted GDF15 exhibited a significantly increased frailty risk (odds ratio = 1.46, 95% confidence interval: 1.01–2.13). Restricted cubic splines analysis revealed a linear dose–response association between DNAm-estimated GDF15 concentrations and frailty. This positive link was persistently evident across diverse subgroups. This cross-sectional study demonstrates a positive correlation linking DNAm-predicted GDF15 concentrations to frailty.

## 1. Introduction

Frailty represents a significant clinical syndrome prevalent among older individuals, characterized by diminished physiological reserve and heightened vulnerability to stressors.^[1]^ Its prevalence ranges from 7% to 25% in community-dwelling older adult cohorts.^[2]^ This multifactorial condition elevates the likelihood of adverse health events, including hospital admissions, functional decline, and mortality,^[3]^ highlighting the critical demand for improved assessment and preventive measures. Current diagnostic approaches, predominantly reliant on clinical evaluations and self-reports,^[4]^ frequently suffer from limited precision and may fail to capture underlying biological pathways. Consequently, an integrative framework is essential for advancing frailty comprehension,^[5]^ particularly concerning epigenetic alterations.^[6]^

Emerging research points to a connection between DNA methylation signatures and frailty, suggesting epigenetic alterations could function as diagnostic markers for this syndrome.^[7,8]^ Notably, the cytokine growth differentiation factor 15 (GDF15), which responds to cellular stress, has attracted interest because of its links to aging processes and frailty phenotypes.^[9]^ Higher circulating GDF15 concentrations show correlation with elevated frailty indices, implying its potential role in reflecting the biological mechanisms driving frailty.^[10,11]^ Additionally, variables like age, gender, ethnicity, and health behaviors such as tobacco use and alcohol intake have been demonstrated to affect both frailty status and GDF15 concentrations, reflecting the multifaceted nature of their interaction.^[12]^

This investigation seeks to clarify the relationship linking DNA methylation-based GDF15 concentrations to frailty, advancing comprehension of this complex phenotype shaped by genetic, environmental, and lifestyle influences. By synthesizing epigenetic information with clinical evaluations, our work aims to refine frailty detection and care strategies for older individuals, ultimately enhancing health outcomes within this susceptible group.^[1]^

Leveraging the National Health and Nutrition Examination Survey (NHANES) database, this research applies weighted logistic regression alongside other analytical techniques to explore connections between DNA methylation (DNAm)-estimated GDF15 and frailty status. NHANES offers a key benefit through its extensive data acquisition, facilitating rigorous examination of health metrics in varied demographic groups. The principal goal is to determine the link between DNAm-derived GDF15 concentrations and frailty occurrence in the geriatric population, underscoring its possible relevance for health surveillance and preventive approaches targeting older adults.

## 2. Methods

### 2.1. Sample population

**Figure 1.**
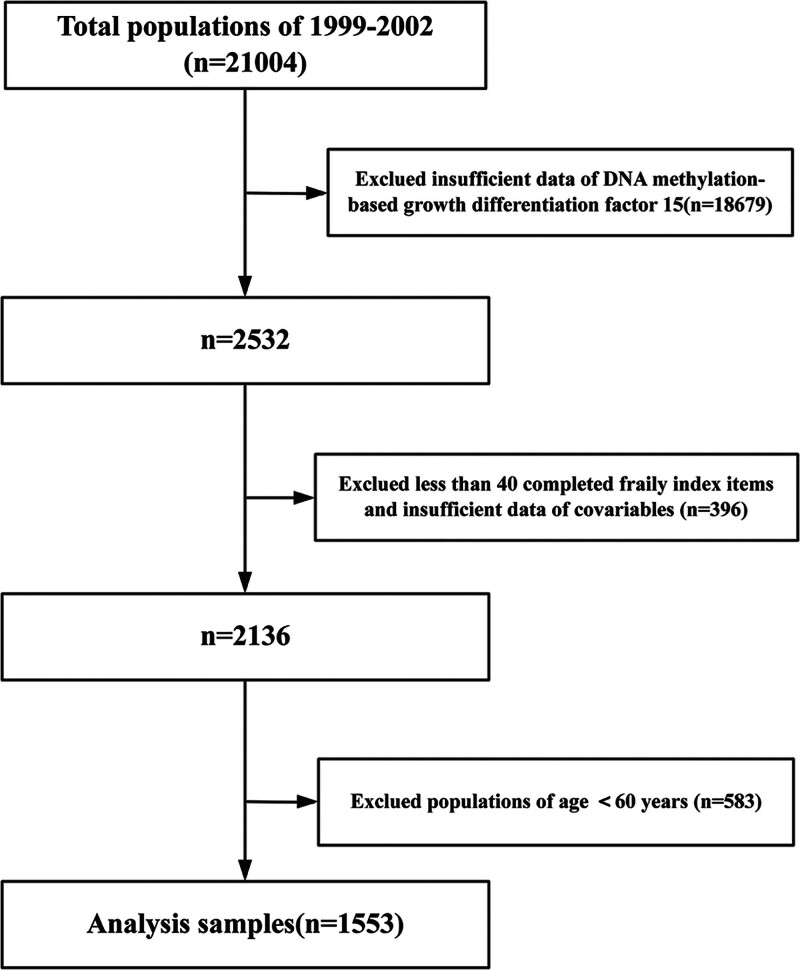
Flow chart of the study participants’ selection process.

This analysis is grounded in data from the NHANES, a nationally representative cross-sectional study systematically compiled by the Centers for Disease Control and Prevention. This program comprehensively tracks the health and nutritional profiles of the U.S. populace.^[13,14]^ Our study concentrated on information from the 1999 to 2002 NHANES cycles, selected as they uniquely offered complete DNA methylation datasets. A total of 21,004 respondents were enrolled during the 1999 to 2002 survey period. Exclusion criteria encompassed: incomplete DNA methylation data (n = 18,679); insufficient frailty index items (<40 completed) or covariate details (n = 396); and age under 60 years (n = 583). Thus, the final analytical cohort comprised 1553 subjects (see Fig. 1). The National Center for Health Statistics Ethics Review Board approved the data acquisition protocol (Protocols #98-12), and written informed consent was secured from all respondents. Furthermore, this study’s methodology and reporting complied with the Declaration of Helsinki ethical principles and followed STROBE Statement recommendations.^[15]^

### 2.2. DNA methylation measurement

Blood specimens for DNA isolation were collected from adults aged ≥50 years participating in the 1999 to 2002 NHANES cycles. The sample represented roughly half of eligible non-Hispanic White individuals chosen randomly, plus all eligible subjects from non-Hispanic Black, Mexican American, other Hispanic, and additional racial/ethnic groups. DNA was extracted from whole blood and stored at ‐80°C. Methylation analysis was performed in Dr Yongmei Liu laboratory at Duke University. Bisulfite conversion adhered strictly to the manufacturer’s instructions; specifically, 500 ng of DNA was processed using Zymo EZ Methylation kit. Converted DNA was then amplified following Illumina’s Infinium assay PCR protocol (16 cycles: 95°C for 30 seconds, 50°C for 60 minutes). Subsequently, 4 µL of bisulfite-treated DNA underwent Illumina’s Infinium HD Methylation protocol, which included overnight (20–24 hours) denaturation/amplification, fragmentation, precipitation, resuspension, and hybridization on EPIC BeadChip v1.0 for 16–24 hours. Post-wash, nucleotide labeling extended the primers. Methylation data acquisition utilized Illumina’s iScan system for BeadChip imaging. For preprocessing NHANES blood specimens, a regression calibration algorithm estimated immune cell proportions from DNAm data. The IDOL probe subset and FlowSorted.Blood.EPIC_ref reference dataset were employed with the “estimateCellCounts2” function to predict cellular composition.^[16–18]^ DNAm-predicted GDF15 values were derived via regression analysis adjusting for chronological age, sex, and pertinent CpG levels.

### 2.3. Assessment of frailty

Adopting the methodology established by Hakeem et al., we employed the frailty index (FI) to quantify frailty severity. This measure encompasses 49 items across multiple domains, including cognitive function, dependency levels, manifestations of depression, comorbid conditions, overall health perception, healthcare utilization, physical function, anthropometric indices, and laboratory parameters.^[19–21]^ Participants were required to complete at least 80% (≥40 items) of the 49 FI components during the eligibility survey. Defect severity was assigned points on a 0 to 1 scale (Supplementary Table S1, Supplemental Digital Content, https://links.lww.com/MD/Q989). The FI value represents the cumulative deficit score for each participant divided by the maximum possible deficit score. Using a validated FI cutoff of 0.21, individuals were categorized into 2 groups: non-frail (FI ≤ 0.21) and frail (FI > 0.21).^[22]^

### 2.4. Assessment of covariates

This investigation accounted for recognized confounders identified in previous studies.^[23]^ Adjusted covariates comprised: Age (continuous variable), sex (male or female).

Race/ethnicity: Non-Hispanic White (NHW), non-Hispanic Black (NHB), Mexican American (MA), and other. Educational attainment: <high school, high school graduate, and >high school. Poverty–income ratio: <130%, 130%–350%, and ≥350%.^[24]^ Smoking status (defined as having smoked ≥100 cigarettes lifetime). Alcohol Intake (defined as consuming ≥12 alcoholic drinks in the previous year).

### 2.5. Statistical analysis

GDF15 concentrations predicted by DNA methylation were stratified into tertiles (T1, T2, and T3). Differences across these tertiles were examined using the chi-square (*χ*^2^) test for categorical variables and one-way analysis of variance (ANOVA) for continuous measures. Continuous data are expressed as mean ± standard deviation. Binary and multi-categorical variables are summarized as frequencies and percentages in Table 1. To evaluate the association linking frailty with DNAm-estimated GDF15, 3 weighted logistic regression models were developed: Model 1: Unadjusted. Model 2: Adjusted for sex, age, and race/ethnicity. Model 3: Adjusted for all covariates listed in Table 1 (excluding frailty). Sensitivity analysis evaluated result stability. Restricted cubic splines (RCS) visualized the dose–response relationships. Subgroup analyses stratified participants by: Age (≤65 or >65 years), sex (male or female), ethnicity (White or non-White), smoking status (no or yes), alcohol consumption (no or yes), potential interactions between DNAm-predicted GDF15 and frailty were tested within the NHANES 1999–2002 cohort. All statistical procedures and visualizations were executed using R software (version 4.3.3; R Foundation for Statistical Computing, Vienna, Austria), applying a significance level of *P* < .05.

**Table 1:** Baseline characteristics of participants according to the tertiles of DNAm-predicted GDF15.

Variables	Total (n = 1553)	T1 (n = 518)	T2 (n = 517)	T3 (n = 518)	*P* value
Gender, n (%)					.44
Male	791 (50.9)	256 (49.4)	275 (53.2)	260 (50.2)	
Female	762 (49.1)	262 (50.6)	242 (46.8)	258 (49.8)	
Age, yr	70.7 ± 7.7	64.1 ± 3.6	70.7 ± 5.8	77.2 ± 6.7	<.001
Race, n (%)					<.001
Mexican American	455 (29.3)	186 (35.9)	168 (32.5)	101 (19.5)	
Other Hispanic	83 (5.3)	24 (4.6)	33 (6.4)	26 (5)	
Non-Hispanic White	654 (42.1)	165 (31.9)	207 (40)	282 (54.4)	
Non-Hispanic Black	316 (20.3)	119 (23)	97 (18.8)	100 (19.3)	
Other race	45 (2.9)	24 (4.6)	12 (2.3)	9 (1.7)	
Education level, n (%)					.085
Below high school	747 (48.1)	234 (45.2)	259 (50.1)	254 (49)	
High school	323 (20.8)	107 (20.7)	95 (18.4)	121 (23.4)	
Above high school	483 (31.1)	177 (34.2)	163 (31.5)	143 (27.6)	
PIR, n (%)					<.001
≤1.3	498 (32.1)	148 (28.6)	175 (33.8)	175 (33.8)	
1.3–3.5	649 (41.8)	195 (37.6)	209 (40.4)	245 (47.3)	
≥3.5	406 (26.1)	175 (33.8)	133 (25.7)	98 (18.9)	
Smoking status (at least 100 cigarettes in life, n (%)					.027
Yes	824 (53.1)	251 (48.5)	280 (54.2)	293 (56.6)	
No	729 (46.9)	267 (51.5)	237 (45.8)	225 (43.4)	
Alcohol intake (at least 12 alcohol drinks per year), n (%)					.144
Yes	944 (60.8)	324 (62.5)	323 (62.5)	297 (57.3)	
No	609 (39.2)	194 (37.5)	194 (37.5)	221 (42.7)	
Frailty n (%)					<.001
No	1032 (66.5)	386 (74.5)	361 (69.8)	285 (55)	
Yes	521 (33.5)	132 (25.5)	156 (30.2)	233 (45)	

DNAm = DNA methylation, GDF15 = growth differentiation factor 15, PIR = family income-to-poverty ratio.

## 3. Results

### 3.1. The baseline features of the participants

This investigation recruited 1553 participants, averaging 70.7 ± 7.7 years in age, with females comprising 762 individuals (49.1%). Applying the frailty index criteria adopted for this research, 33.5% of the cohort was categorized as frail. Table 1 details the overall demographic and clinical profile of the study population. Statistical analyses revealed significant variations across the DNAm-predicted GDF15 tertile groups concerning age, race, smoking status, the family income-to-poverty ratio, and frailty status. Conversely, sex distribution, educational attainment, and alcohol consumption showed no statistically significant differences.

### 3.2. The relationship between DNAm-predicted GDF15 and frailty

**Figure 2.**
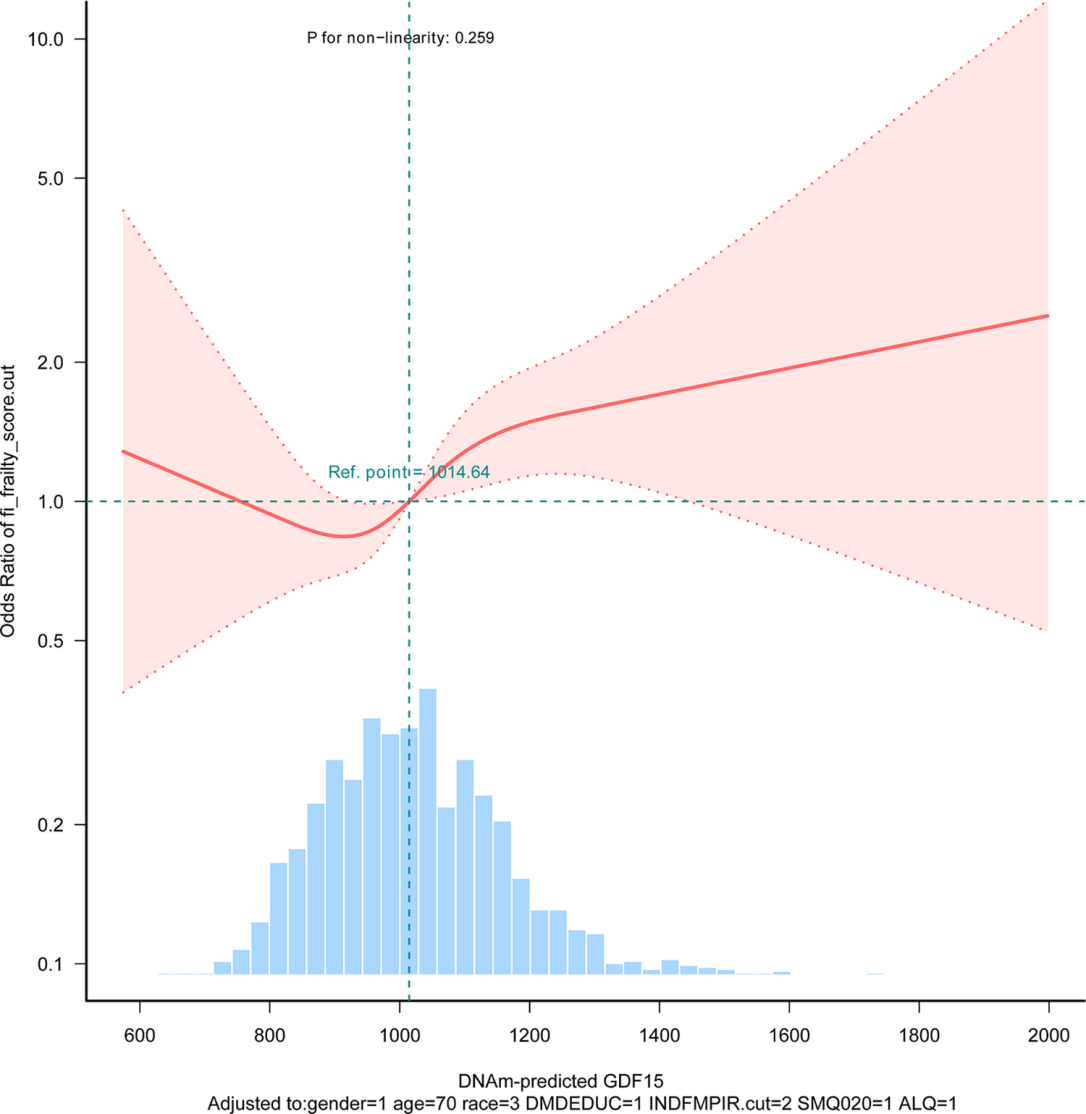
RCS analysis for the association between DNAm-predicted GDF15 and risk of frailty. DNAm = DNA methylation, GDF15 = growth differentiation factor 15, RCS = restricted cubic spline.

To examine the relationship between DNAm-predicted GDF15 and frailty, 3 logistic regression analyses were conducted. Table 2 displays the odds ratios (OR) and corresponding 95% confidence intervals (CI) quantifying this association. In the initial unadjusted model (Model 1), treating DNAm-predicted GDF15 as a continuous variable yielded an OR of 1.29 (95% CI: 1.2–1.39, *P* < .001). When categorized, the second tertile (T2) demonstrated an OR of 1.26 (95% CI: 0.96–1.66, *P* < .001), and the third tertile (T3) exhibited a significantly higher OR of 2.39 (95% CI: 1.84–3.11, *P* < .001), with a statistically significant trend observed (*P* for trend < .001). Model 2 incorporated adjustments for age, sex, and ethnicity, resulting in a decreased OR of 1.18 (95% CI: 1.07–1.3, *P* = .001) for the continuous GDF15 measure. Correspondingly, the OR values for T2 and T3 declined to 1.05 (95% CI: 0.78–1.43, *P* < .001) and 1.68 (95% CI: 1.17–2.43, *P* < .001), respectively, while maintaining a significant trend (*P* for trend < 0.001). The comprehensively adjusted Model 3, which included additional covariates (poverty–income ratio, education level, smoking status, alcohol consumption), showed a further reduction in the continuous GDF15 OR to 1.14 (95% CI: 1.03–1.26, *P* = .014) The categorical analysis revealed ORs of 0.96 (95% CI: 0.7–1.31, *P* < .001) for T2 and 1.46 (95% CI: 1.01–2.13, *P* < .001) for T3, with the trend remaining statistically significant (*P* < .001). Additionally, RCS were utilized to graphically depict the association. After adjusting for all covariates specified in Model 3, the RCS analysis revealed a linear relationship between DNAm-predicted GDF15 and frailty (Fig. 2, *P* for nonlinearity = .259).

**Table 2:** Association between DNAm-predicted GDF15 and frailty in older adults.

	Model 1	Model 2	Model 3
	OR (95% CI) *P* value	OR (95% CI) *P* value	OR (95% CI) *P* value
DNAm-predicted GDF15 as continuous variable			
Per 100 units	1.29 (1.2, 1.39) < .001	1.18 (1.07, 1.3) .001	1.14 (1.03, 1.26) .014
DNAm-predicted GDF15 as categories variable			
T1	1.0	1.0	1.0
T2	1.26 (0.96, 1.66) < .001	1.05 (0.78, 1.43) <.001	0.96 (0.7, 1.31) <.001
T3	2.39 (1.84, 3.11) < .001	1.68 (1.17, 2.43) <.001	1.46 (1.01, 2.13) <.001
*P* for trend	<.001	<.001	<.001

Model 1: Not adjusted any covariates; Model 2: Adjusted age, sex and race; Model 3: Adjusted age, sex, race, PIR, education level, smoking status, and alcohol status.

CI = confidence interval, DNAm = DNA methylation, GDF15 = growth differentiation factor 15, OR = odds ratio, PIR = poverty–income ratio.

### 3.3. Subgroup analysis

**Figure 3.**
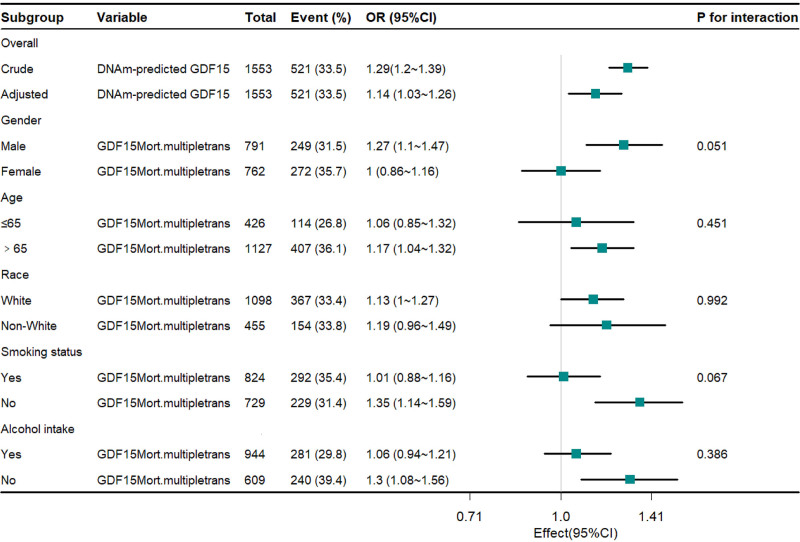
The forest plot showed the correlation between DNAm-predicted GDF15 and frailty (OR 95%). DNAm = DNA methylation, GDF15 = growth differentiation factor 15, OR = odds ratio.

o evaluate the robustness of our findings, subgroup analyses were performed across diverse populations (Fig. 3). Results stratified by multiple variables, including age, sex, race, smoking status, and alcohol intake, revealed no statistically significant interactions (all *P* for interaction > .05). Crucially, the positive association linking DNA methylation-predicted GDF15 levels with frailty persisted consistently within all examined subgroups.

## 4. Discussion

This research tackles the pressing challenge of frailty among older adults, delineating a significant link between DNAm-predicted GDF15 levels and frailty status. Diverging from prior investigations predominantly reliant on clinical frailty evaluations, our work pioneers the application of DNA methylation as a predictive biomarker, offering fresh insights into the associated biological pathways. These results emphasize the imperative necessity for developing potent health management approaches targeting the older adults, especially for early identification of individuals susceptible to frailty.

Leveraging data from the NHANES, our investigation uncovered a strong link between heightened DNAm-predicted GDF15 concentrations and elevated frailty risk. Multiple logistic regression analyses consistently confirmed that rising GDF15 levels correlate with a higher probability of frailty, supporting the potential utility of GDF15 as a promising biomarker for the early detection of frailty among seniors. These findings advance the comprehension of frailty pathogenesis and underscore the imperative to incorporate molecular indicators into standard geriatric health evaluations.

This investigation identifies a marked positive relationship linking DNA methylation-derived GDF15 concentrations to frailty, proposing GDF15 as a candidate biomarker for evaluating frailty. Existing literature corroborates this link; 1 investigation, for instance, observed that increased GDF15 concentrations correlated with heightened frailty probability, underlining GDF15’s predictive value in aging cohorts.^[25]^ Research involving 800 subjects further established that elevated GDF15 levels corresponded with diminished physical function and greater frailty prevalence, solidifying the concept of GDF15 as a key element in frailty evaluation.^[26]^ These prior investigations resonate with our outcomes, accentuating GDF15’s significance in deciphering frailty pathophysiology. However, contrasting findings exist. A cohort analysis of 500 seniors, for example, detected no meaningful link between GDF15 concentrations and frailty status, implying that additional determinants might independently affect frailty development.^[27]^ Such inconsistencies may stem from variations in participant numbers, demographic profiles, methodological strategies (including specific frailty measurement instruments), as well as differences in environmental exposures and genetic backgrounds across populations. Collectively, the recurrent evidence connecting GDF15 to frailty across multiple studies underscores its biomarker promise, whereas contradictory data illustrate the intricacy of frailty as a condition influenced by numerous factors. Additional inquiry is necessary to clarify the fundamental biological processes and validate GDF15’s applicability in clinical practice.

Utilizing a logistic regression framework, this research examined the linkage between DNAm-predicted GDF15 concentrations and frailty, uncovering a significant exposure–outcome linkage persisting after extensive covariate adjustment. Three separate weighted logistic models were constructed to thoroughly investigate the connection between the exposure (GDF15 concentrations) and outcome (frailty status). The initial model, without covariates, yielded an OR of 1.29 (95% CI: 1.2–1.39, *P* < .001) for GDF15 treated continuously, signifying a robust positive association with frailty. Subsequent models incorporating age, sex, and ethnicity showed a reduced OR of 1.18 (95% CI: 1.07–1.3, *P* = .001) for continuous GDF15. Analysis using tertiles revealed that the highest tertile (T3) retained a statistically significant OR of 1.46 (95% CI: 1.01–2.13, *P* < .001). The temporal stability of these associations across sequential models underscores result robustness, evidenced by a persistently significant trend (*P* for trend < .001) in the final adjusted model. Complementary RCS analyses verified a linear dose–response relationship between GDF15 concentrations and frailty probability, providing further confirmation of the logistic regression findings. Employing diverse analytical strategies strengthens the credibility of the conclusions, as they collectively demonstrate, from multiple angles, a positive linear relationship between GDF15 exposure and frailty outcome. This methodological triangulation not only reinforces evidence for GDF15’s role in frailty pathogenesis but also accentuates the necessity of employing varied statistical models to elucidate intricate biological associations within epidemiological research. The results imply GDF15’s potential as a frailty biomarker, justifying deeper exploration into its mechanistic contributions to aging processes and associated health trajectories.

This investigation demonstrates a significant association between DNA methylation-predicted GDF15 concentrations and frailty, implying a plausible connection between cellular senescence processes and inflammatory signaling within the aging framework. Specifically, heightened GDF15 levels, inferred from DNA methylation signatures, exhibit correlation with increased frailty severity—a state marked by diminished physiological resilience and heightened susceptibility to stress. This association implicates senescent cells, recognized secretors of pro-inflammatory cytokines contributing to the senescence-associated secretory phenotype, in driving the frailty phenotype. Notably, the research underscores the relevance of particular cellular subtypes, including senescent endothelial cells, which might display modified reactions to therapeutic strategies targeting frailty mitigation. Moreover, senescent fibroblasts, identified in related work, constitute another critical subtype within the inflammatory environment linked to aging and frailty. Consequently, subsequent investigations ought to prioritize identifying key transcription factors governing these cellular subtypes. Elucidating their functional contributions could illuminate targeted therapeutic approaches to modulate senescence effects and ameliorate health outcomes among frail populations. Discovering these regulators may additionally unveil novel biomarkers for frailty assessment and inform interventions designed to bolster resilience in older adults.

## 5. Limitations

While these findings advance our understanding of epigenetic frailty biomarkers, several methodological considerations warrant Acknowledgments. This investigation encompasses noteworthy observations on the relationship linking DNAm-predicted GDF15 concentrations to frailty within a cohort of 1553 individuals, among whom one-third (33.5%) exhibited frailty status. Nevertheless, several constraints warrant recognition. Primarily, the omission of bench validation procedures precludes confirmation of methylation data accuracy and associated biological consequences. Moreover, although participant numbers are substantial, the cross-sectional methodology constrains causal interpretation. The absence of external validation analyses additionally questions result applicability across diverse demographics. Potential technical artifacts stemming from aggregated dataset utilization might further introduce confounding variability influencing outcome interpretation.

## 6. Conclusion

Collectively, this research delineates a dose–response association between DNA methylation-derived GDF15 concentrations and frailty severity, positioning GDF15 as a candidate biomarker for frailty evaluation. These discoveries carry considerable translational weight, establishing foundations for mechanistic investigations into GDF15-frailty pathways. Furthermore, incorporating machine learning algorithms to process methylation signatures substantially augments predictive accuracy in frailty identification, enabling refined clinical protocols for early detection and management of high-risk individuals. Subsequent investigations ought to prioritize prospective cohort designs and clinical verification studies to consolidate evidence regarding GDF15’s pathophysiological significance and viability as a treatment target.

## Acknowledgments

We would like to thank all NHANES participants and staff. We also thank Free Statistics team for providing tools of value for data analysis. We appreciate Dr Jie Liu (People’s Liberation Army of China General Hospital, Beijing, China) for his contribution to statistics and study design consultations.

## Author contributions

**Conceptualization:** Zou Meisheng.

**Formal analysis:** Zou Meisheng.

**Funding acquisition:** Zou Meisheng.

**Investigation:** Wu Suhong.

**Methodology:** Wu Suhong.

**Project administration:** Wu Suhong.

**Resources:** Zou Meisheng, Wu Suhong.

**Software:** Zou Meisheng, Wu Suhong.

**Supervision:** Zou Meisheng, Wu Suhong.

**Validation:** Wu Suhong.

**Visualization:** Wu Suhong.

**Writing – original draft:** Zou Meisheng.

**Writing – review & editing:** Zou Meisheng, Wu Suhong.

## Supplementary Material


